# Identifying potential differences in cause-of-death coding practices across Russian regions

**DOI:** 10.1186/s12963-016-0078-0

**Published:** 2016-03-22

**Authors:** Inna Danilova, Vladimir M. Shkolnikov, Dmitri A. Jdanov, France Meslé, Jacques Vallin

**Affiliations:** Max Planck Institute for Demographic Research, Konrad-Zuse-Strasse 1, 18057 Rostock, Germany; National Research University Higher School of Economics, Myasnitskaya St. 20, 101000 Moscow, Russia; New Economic School, Novaya St. 100, Skolkovo, 143026 Moscow, Russia; Institut national d’études démographiques, Blvd. Davout 133, 75020 Paris, France

**Keywords:** Causes of death, Coding, Mortality statistics, Validation, Russia

## Abstract

**Background:**

Reliable and comparable data on causes of death are crucial for public health analysis, but the usefulness of these data can be markedly diminished when the approach to coding is not standardized across territories and/or over time. Because the Russian system of producing information on causes of death is highly decentralized, there may be discrepancies in the coding practices employed across the country. In this study, we evaluate the uniformity of cause-of-death coding practices across Russian regions using an indirect method.

**Methods:**

Based on 2002–2012 mortality data, we estimate the prevalence of the major causes of death (70 causes) in the mortality structures of 52 Russian regions. For each region-cause combination we measured the degree to which the share of a certain cause in the mortality structure of a certain region deviates from the respective inter-regional average share. We use heat map visualization and a regression model to determine whether there is regularity in the causes and the regions that is more likely to deviate from the average level across all regions. In addition to analyzing the comparability of cause-specific mortality structures in a spatial dimension, we examine the regional cause-of-death time series to identify the causes with temporal trends that vary greatly across regions.

**Results:**

A high level of consistency was found both across regions and over time for transport accidents, most of the neoplasms, congenital malformations, and perinatal conditions. However, a high degree of inconsistency was found for mental and behavioral disorders, diseases of the nervous system, endocrine disorders, ill-defined causes of death, and certain cardiovascular diseases. This finding suggests that the coding practices for these causes of death are not uniform across regions. The level of consistency improves when causes of death can be grouped into broader diagnostic categories.

**Conclusion:**

This systematic analysis allows us to present a broader picture of the quality of cause-of-death coding at the regional level. For some causes of death, there is a high degree of variance across regions in the likelihood that these causes will be chosen as the underlying causes. In addition, for some causes of death the mortality statistics reflect the coding practices, rather than the real epidemiological situation.

## Background

Data on mortality by causes of death are important for monitoring epidemiological patterns. These data, which are widely used in demographic and medical research, often provide crucial information for identifying public health problems and developing health care strategies. However, the usefulness and the interpretability of mortality data depend largely on their quality, which varies between countries [1–3].

The quality of cause-of-death reporting in a certain country or territory is often assessed by examining the prevalence of obvious flaws in cause-specific mortality data (such as the use of unspecified or “garbage” causes, or the violation of the logical correspondence between the causes of death and the age or sex of the deceased) [1, 4]. But even if the prevalence of such obvious problems is shown to be moderate, the quality of cause-of-death reporting can be considered imperfect; even if these problems are not immediately apparent, it is possible that certain causes of death are being systematically misclassified [5–11], and that the cause-specific mortality data being generated are therefore of limited utility for the purposes of public health decision-making and research.

Although the International Classification of Diseases (ICD) manuals provide clear and detailed instructions on the coding process, for a variety of reasons the actual coding practices in a country may not follow these rules. Studies that have compared mortality from specific diseases across countries have found that coding practices can vary substantially, and that this lack of consistency has the potential to distort the comparability of the cause-specific mortality data for different populations [12–18]. Medical concepts and coding practices may also vary within a single country, especially if it has a large and diverse population spread over a vast territory. Thus, the consistency of cause-specific mortality data at the subnational level also determines the usability and the interpretability of national and subnational cause-of-death statistics [19, 20].

As Russia covers a very large territory, mortality levels – as well as general socioeconomic, ethno-cultural, and climatic conditions – vary substantially across the country’s regions. In 2013, life expectancy at birth for the Russian population as a whole (both sexes) was 70.8 years. The regional disparities for the same year were substantial: the standard deviation across regions was 2.6 years and the maximum vs. minimum range exceeded 17 years. Reliable data on cause-specific mortality at the subnational level can help to explain the origins of health inequality in Russia, and may prove useful for designing interventions to reduce it.

### The Russian system for cause-of-death diagnostics and coding

The World Health Organization (WHO) has rated the coverage and the quality of cause-of-death mortality data in Russia as “medium” [21]. The Russian system for registering and coding deaths is characterized by almost full coverage of civil registration of the underlying causes of death (estimated completeness in 2006–2012 was 99 %) [22]. Moreover, the share of deaths for which post-mortem autopsies are performed is larger in Russia than in many other countries [23, 24]. However, recent studies on the quality of Russian cause-specific mortality statistics have shown that a large proportion of deaths in Russia are assigned to the ICD codes for various ill-defined and “unspecified” conditions. These codes provide poor information for the purposes of developing health policy [25–27]. In particular, N. Gavrilova and co-authors have made the claim that since the 1990s, there has been a general deterioration over time in the quality of cause-specific mortality statistics in Russia [26]. One piece of evidence that supports this assertion is the increase in the number of deaths in which the deceased’s identity could not be established; this trend was observed throughout the 1990s and the first half of the 2000s [28]. In 2005, an approximate age of death could not be specified for 0.76 % of all deaths in Russia (1.15 % for males and 0.31 % for females), even after a forensic autopsy. Since 2005, this trend has reversed, and by 2013 the share of such cases had been reduced to 0.27 % (0.42 % for males and 0.11 % for females).

Before 1999, detailed ICD was not used in Russia. The Central Statistical Office of the USSR developed brief Soviet Classifications that were roughly based on contemporary versions of the ICD. The Classification of 1981, which was modified in 1988 (hereinafter SC-1988), was the last Soviet Classification, and was in use until 1998. The SC-1988’s list of causes of death consisted of 184 aggregated items based on all of the codes of the ICD-9 (plus 10 items for the double classification of external causes of death by the character of the injury).

Russia implemented the ICD-10 in 1999, and since then all death certificates issued in the country must be filled in with the original ICD-10 codes. Even though all medical death certificates have four-digit ICD-10 codes indicating the causes of death, data at this level of detail are unavailable for research purposes. The Russian State Statistics Service (Rosstat) publishes information on causes of death in aggregate form only. In these published data tables, deaths are tabulated in accordance with the Russian Abridged Classification (hereinafter RC-1999) launched in 1999, which consists of 234 items (plus 10 additional items for the double classification of external causes of death) that correspond to groups of detailed ICD-10 codes. In the routine data tables, the age of death is given in categories: 0, 1–4, 5–9, 10–14, …, 85+. In most cases, researchers have access to aggregate data only. These aggregate data tables are provided to the WHO by the Rosstat and the Russian Ministry of Health.

For Russia, the transition to the ICD-10 in 1999 represented not just a move to a new cause-of-death classification, but also entailed changes in the basic principles of coding and gathering information about the causes of death [29]. Before 1999, medical professionals in Russia had no responsibility to assign codes to causes of death. Their main duty was to fill in the medical death certificate by writing down the sequence of medical causes that contributed to the death. These medical death certificates were then submitted by the decedent’s relatives or by the responsible institution to the respective district office of the Registration of Acts of Civil Status (ZAGS), a government body that was (and still is) responsible for the civil registration of deaths and for issuing civil death certificates. A civil death certificate was (and still is) needed for burial and for legal purposes. While the medical death certificate specified the cause of death in detail, the civil death certificate (the document that is given to the relatives as a final document confirming the death event) did not contain any information on the cause of death. The cause-of-death information was excluded from this document starting in 1997. The district offices of ZAGS passed the medical death certificates received from the decedent’s relatives to the Regional Statistics Services, where trained statisticians coded the underlying cause of death according to the contemporary version of the Soviet Abridged Classification. The corresponding cause-of-death data were then computerized and sent to the Central Statistical Administration.

The data processing system has changed since 1999, with one of the main differences being that the coding procedure now takes place not at the end of the cycle described above, but at the very beginning. Medical professionals in Russia are now responsible not only for certifying the death, making the diagnosis, and indicating the sequence of the causes that contributed to death; but also for coding the death in accordance with the ICD-10 rules. There is no centralized and/or automated coding system to assist the medical professionals in choosing and coding the underlying causes. The statisticians in the Regional Statistics Services are now only responsible for checking the ICD codes for obvious mistakes, and for aggregating these codes into RC-1999 items.

The Russian system of cause-of-death statistics is thus highly decentralized. Since 1999, each medical practitioner in charge of issuing death certificates constitutes a separate coding unit. Such a system may have certain advantages for Russia, as the country has a huge territory and a large population distributed very unevenly across space. However, this system may also result in coding discrepancies across territorial units. We should note here that although the coding process in Russia is performed at the level of individual medical workers, it is likely that some territorial “schools” of coding exist. In most of the Russian regions, the majority of medical death certificates are issued by a limited number of parties: the physicians responsible for coding in a few large hospitals, the autopsy departments in these large hospitals, and several forensic examination bureaus (in 2012, 50.6 % of all deaths were subject to autopsy). Thus, the coding practices in a limited number of institutions may largely determine each region’s approach to coding. These institutions are in turn accountable to the regional Ministry of Health, and are expected to follow the decrees and instructions issued by this authority. All of the practitioners working in the medical institutions that are in charge of filling in the medical death certificates are expected to follow the same guidelines. We can therefore expect that the medical practitioners in a given region will tend to use similar approaches to diagnosis and coding.

### Prior research on the topic

There have been a number of studies that have addressed the quality of cause-specific mortality statistics in the USSR and Russia (Table 1). First, there are a few audits conducted in the USSR to assess the accuracy of death certification. The general atmosphere of conspiracy and the desire to avoid announcing unfavorable mortality trends in the USSR led to the keeping data on all-cause and cause-specific mortality in secrecy, and to scarcity of statistical publications on mortality. The three studies shown in Table 1 were identified by a team of French and Russian demographers in the 1990s [30]. These studies had similar designs, and were based on a re-inspection of samples of medical death certificates. Following their analysis of the results of the three surveys, the researchers concluded – despite previous expectations to the contrary – that there was no evidence of significant overestimation of deaths from all cardiovascular diseases combined in the USSR and Russia. Specifically, they found that even though the error rates were high for specific circulatory conditions, these problems were compensated for within the ICD chapter “circulatory diseases.” When the researchers looked at the other groups of causes, they found the largest shares of diagnosis and coding errors for digestive and respiratory diseases.

**Table 1:** Prior research on the topic

Studies	Time and location	Purpose	Data and methods	Findings
*Re*-*coding studies*				
1. Bystrova (1965); 2. Bednyi et al. (1980,1981); 3. Ovcharov, Bystrova (1982) *studies cited by Meslé et al. (1996) [31]	1. Cities of Tula, Novomoskovsk, Tambov, Michurinsk in the early 1960s 2. “Different regions of Russia” (not specified), 1979. 3. Belarus and Turkmenistan, 1981–1982.	To assess the accuracy of cause-of-death diagnostics and coding in the USSR.	- Re-inspection of medical death certificates. - Number of deaths in the sample, age and sex distributions not described.	- Against a widely held view, no evidence was found of substantial overestimation of cardiovascular mortality in the USSR and Russia. - High percentages of false diagnoses for certain circulatory causes with compensation within the same ICD chapter were found. The total for the whole class was trustworthy. - The lowest percentage of misclassified cases was found for neoplasms and external causes; the highest was found for digestive and respiratory diseases.
Shkolnikov, Chervyakov et al. (2000) [34]	Cities of Udmurt Republic, 1998-1999	To inspect the quality of cause-of-death coding under the conditions of the “mortality crisis” of the 1990s.	- Analysis of 1,023 medical death certificates of males aged 20–55. - Re-coding of 782 deaths by an independent and experienced expert.	- For 98 % of deaths the ICD chapter of the initial underlying cause coincided with the cause specified by the expert. - The level of concordance decreased at the lower level of cause-of-death aggregation. - The most significant misclassification was found within the chapter of circulatory diseases and within the chapter of external causes of death.
Vaysman (2013) [40]	Tula obl., 2008, 2012	To analyze the accuracy of the reporting of causes of death in the Tula region.	- Re-coding of a sample of 148 medical death certificates.	- Share of deaths was attributed to circulatory diseases decreased from 66.2 % to 56.8 % after re-coding. - Share of deaths from endocrine disorders, neoplasms, digestive system disorders increased.
*Studies based on inspection of cause*-*specific mortality trends*				
1. Meslé et al. (1996) [31] 2. Shkolnikov et al. (1996) [32, 33]	1. Russia as a whole, 1965–1994 2. Russia as a whole, 1970-1993	To analyze the different aspects of mortality for a long-term period, and to explore the components of the health crisis in Russia (USSR).	- The method of reconstruction [45, 46, 58]. - Visual inspection of cause- and age-specific mortality trends.	- Overestimation of the cardiovascular deaths total was not confirmed. - CVD mortality may even be underestimated among those aged 80+ due to excess use of “senility.” - Some misclassification found for several causes within the class of circulatory diseases.
*Analyses of post*-*mortem reports*				
Shkolnikov et al. (2002) [35]	Izhevsk, 1998-1999	To determine whether deaths from alcohol poisoning were being misclassified as cardiovascular deaths.	- 309 deaths of males aged 20–55 with necropsy records were checked for blood alcohol concentration (BAC).	- No evidence that alcohol poisonings were misclassified as cardiovascular deaths was found. - Among 10 deaths (3.2 % of the sample) with potentially lethal BAC (>4 g/L) seven were coded as accidental poisonings, two as suicides, one as a traffic accident. - No deaths with BAC > 4 g/L were coded as cardiovascular diseases.
Zaridze et al. (2009) [37]	Barnaul, 1990-2004	To determine the role of alcohol in unusual fluctuations in mortality in Russia.	- Inspection of cause-specific mortality trends in Russia in 1991–2006. - BAC values and official diagnoses for 24,836 forensic autopsies in 1990–2004 in the city of Barnaul.	- A post-mortem potentially lethal BAC (>4 g/L) found for 14 % of deaths of males aged 35–69 officially recorded as deaths from cardiovascular causes. - During the period of large mortality fluctuations in Russia in the 1990s, mortality from myocardial infarction (MI) did not follow the fluctuating trends of other cardiovascular causes. A low percentage of the deaths with lethal BAC levels were found among MI deaths. - Abrupt changes in the death rates from many circulatory causes may be caused by the misclassification of alcohol poisonings.
Leon et al. (2010) [36]	Izhevsk, 2003-2005	To find a link between alcohol and mortality among men of working age.	- Inspection of cause-specific mortality trends in Russia in 1980–2007. - Case–control study (1750 cases – deaths of males aged 25–54; 1750 controls – live men) with interviews. - Deaths subjected to forensic autopsy checked for BAC.	- Criticism of the results of Barnaul study (Zaridze et al., 2009): 1) mortality from cerebrovascular diseases fluctuated significantly in the 1980s and the 1990s, in tandem with mortality from alcohol causes; yet the percentages of deaths with BAC >4 g/L were very low for this group of causes in both Barnaul and Izhevsk. These findings contradict the evidence from the Barnaul study; 2) during the years immediately following the transition to the RC-1999 there was an artificial conflation of mortality from ischemic and non-ischemic heart diseases in Barnaul. Thus, the mortality trends in Barnaul were not nationally representative. - In Izhevsk, only 5 % of deaths certified as deaths from circulatory diseases showed BAC higher than 4 g/L at post-mortem autopsy.
Sidorenkov et al. (2011) [24]	Arkhangelsk, 2008-2009	To determine whether deaths from alcohol poisoning were misclassified as cardiovascular deaths.	- All deaths at ages 30–70 from cardiovascular diseases subjected to forensic autopsy checked for BAC.	- No evidence of alcohol poisonings being misclassified as cardiovascular deaths was found. - Only six cases of deaths with BAC higher than 4 g/L were certified as cardiovascular deaths.
*Studies analyzing information contained in medical death certificates*				
Gavrilova et al. (2008) [27]	Russia as a whole, 1991–2005; Kirov and Smolensk regions, the city of Moscow, 2003	To investigate which causes of death are hidden under ill-defined conditions.	- Descriptive statistical analysis of mortality from ill-defined conditions in Russia. - Analysis of various types of information from medical death certificates.	- The instructions on death certification in Russia encourage medical practitioners to use unspecified diagnosis in medical death certificates. - A significant fraction of deaths from ill-defined conditions at working age are deaths from external causes (including violence) hidden under other diagnoses.
Lopakov (2011) [39]	Kaluga region, 2002	To find mistakes in medical death certificates.	- Analysis of 419 medical death certificates.	- Mistakes in medical death certificates were found. - The shares of “other” and “unspecified” diagnoses were too high.
Roschin et al. (2013) [41]	- Three hospitals in the Moscow region, 2002 - Tver, Tula, and Kaluga regions, 2002	To assess the accuracy of reporting diabetes in medical death certificates.	- Comparison of medical death certificates and medical records for individuals who died in hospitals. - Analysis of the information reported in medical death certificates	- While 25 % of the deceased in hospitals had diabetes, this diagnosis was not specified in death certificates (other than in a few cases in which diabetes was selected as an underlying cause of death). The frequency of the reporting of diabetes in medical death certificates did not correspond to its real prevalence in the population.
*Studies examining the comparability of cause*-*of*-*death mortality data reporting across regions*				
Pridemore (2003) [42]	Russia as a whole 1987–1998; 78 regions of Russia 1994-1998	To evaluate the homicide reporting in Russia.	- Comparison of two sources of homicide estimates in Russia: data from the vital statistics and data from the Ministry of the Interior.	- Disparities across regions in the reporting of homicides were found in the mortality and crime data. - In the majority of regions (66 of 78) the number of homicides in the vital statistics was higher than the number in the crime statistics, though the magnitude of the difference varied. - Opposite ratios were observed in 12 regions.
Nemtsov (2003) [43]	77 regions of Russia, 1990-2001	To estimate alcohol-related mortality in the Russian regions.	- Analyzing the mortality from alcohol-related causes in regions. - Comparison of mortality levels from alcohol poisonings and alcohol psychoses across the regions.	- Among all alcohol-related causes of death acute alcohol poisonings had the highest variability across regions. - The mortality levels and the dynamics of the alcohol poisonings and alcohol psychoses did not correspond to each other in many regions; this finding contradicts the current understanding of the link between these causes, and can thus be regarded as a statistical artifact.

When mortality data for Russia were again made available for research and publication at the end of the 1980s, researchers showed a strong interest in investigating various aspects of Russian mortality, and the issue of the validity of cause-of-death data in particular.

The first and the most comprehensive attempt to analyze different aspects of Russian mortality for a longer time period was the project by the French-Russian team mentioned above [30–32]. The outcomes of this project, which were published in 1996, included observations about the quality of cause-specific mortality statistics in Russia (USSR). Certain indirect findings on the validity of cause-of-death data in Russia were based on a visual inspection of cause-specific mortality trends. The analysis was also unable to confirm that there was an overestimation of entire class of cardiovascular diseases in Russia. Indeed, the results indicated that mortality from cardiovascular diseases might have even been underestimated among the elderly. A special decree by the Soviet Ministry of Health in 1989 had resulted in a massive artificial transfer of deaths from the chapter “diseases of the circulatory system” to the ICD chapter “symptoms, signs, and ill-defined conditions.” Moreover, in line with the results of the comparison of the three Soviet surveys, some misclassification was discovered among the causes related to cardiovascular diseases. In particular, the analysis found that Soviet (and later Russian) coding practices tended to assign the excess number of deaths to atherosclerotic heart disease, which resulted in an underestimation of mortality from other ischemic and non-ischemic heart diseases.

Like the earlier surveys of the Soviet era, subsequent studies that assessed the quality of Russian cause-of-death data used direct techniques. Two of these studies were within the framework of the Udmurt and the Izhevsk studies, which were conducted in the Udmurt Republic and its capital, the city of Izhevsk. The aim of these studies was to identify the reasons for the high premature male mortality rates in Russia, and specifically to clarify the link between premature male mortality and hazardous alcohol drinking. The research included the hypothesis that deaths from acute alcohol poisoning may have been misattributed to circulatory diseases. Based on necropsy records and information obtained from medical files, medical experts checked whether the officially recorded underlying cause of death in the medical death certificates was credible. The findings indicated that the ICD chapter assignments in the cause-specific mortality statistics were quite reliable, but that the incidence of misattribution was higher when the recorded cause and the actual cause were in the same chapter [33]. The hypothesis that a significant fraction of deaths from acute alcohol poisoning were being hidden behind the mask of cardiovascular disease was not confirmed [33–35].

Other studies that investigated the possibility that deaths from acute alcohol poisoning were misclassified as deaths from circulatory diseases were also carried out in two other Russian cities, Barnaul [36] and Arkhangelsk [23], by other groups of researchers.

The authors of the Barnaul Study argued that the abrupt changes in the death rates from many circulatory causes (especially from other forms of ischemic heart disease) in the 1990s were caused by the misclassification of deaths from alcohol poisonings. In particular, they based this hypothesis on their finding that between 1990–2004 in Barnaul, the post-mortem blood alcohol concentration (BAC) was lethal (>4 g/L) for 14 % of the autopsied deaths of males aged 35–69 that were officially recorded as deaths from cardiovascular causes. However, these results contradict the outcomes of the Arkhangelsk study and of the earlier Izhevsk study. The authors of the Arkhangelsk study inspected death certificates issued in this city between January 2008 and August 2009 and found no cases in which the death of a man aged 30–49 with BAC > 4 g/l was certified as circulatory disease [23]. The results for Izhevsk indicated that 0 % of deaths of males aged 20–55 in the years 1998–1999 [34] and 5 % of deaths of males aged 25–54 in the years 2003–5 [35] with BAC > 4 g/l were recorded as deaths from circulatory disease.

It is interesting to note that studies that had similar designs, but were conducted in three different cities, produced such a wide range of results. The large discrepancies in the findings of these studies could be at least partly attributable to differences in the approaches to the certification of causes of death in different sites in Russia.

Studies that specifically addressed the issue of the possible misattribution of different causes of death were also conducted in a few other regions of Russia [26, 37–40]. These studies relied primarily on the examination of medical death certificates. In most of these investigations, researchers tried to check whether the underlying cause of death was reported correctly by consulting other information presented in the death record (such as the immediate and contributing causes of death and the place of death). In addition, some researchers used the medical files of the deceased to check the diagnosis [38, 40].

Studies based on the re-inspection of death certificates were performed in only a very few Russian regions and at a few points in time. While it appears that these studies accurately reported the types and the origins of miscoding, it is still not clear to what extent specific regional findings can be generalized to the national level. Thus, these studies do not provide us with any conclusive insights into how the quality of coding varies across regions in Russia.

A comprehensive evaluation of the comparability of cause-of-death mortality data reporting by Russian regions has not yet been conducted. We are aware of only two papers that specifically examined regional peculiarities in the coding of some specific causes of death in Russia. The first is the 2003 study conducted by W. Pridemore that investigated the comparability of two sources of homicide estimates in Russia: data from the vital statistics registration system and data from the Ministry of the Interior [41]. The results showed that there were certain disparities across regions in the reporting of homicides in the mortality and crime data. The second study on coding discrepancies across Russian regions, by A. Nemtsov, examined spatial-temporal variations in alcohol-related mortality in Russia [42]. Comparing mortality levels from acute alcohol poisonings and alcohol psychoses, Nemtsov showed that the mortality levels and the dynamics of these two causes did not correspond to each other in many regions; this finding contradicts our current understanding of the link between these causes, and can be regarded as a statistical artifact. Although the studies by Pridemore and Nemtsov exclusively examined the statistics on, respectively, homicides and alcohol-related causes, they provide us with some important insights into the different regional approaches to cause-of-death reporting.

### Specific objective of the current study

Here we present the first study that systematically addresses the problem of the comparability of cause-specific mortality statistics across Russian regions. Our purpose in this study is to use the indirect tools and the limited data available to provide a snapshot of the quality of cause-of-death mortality statistics in Russia at the regional level. Our overview offers some instantaneous, easy-to-interpret results, and can also serve as a starting point for more in-depth investigations. Specifically, we aim to:

- evaluate the regional cause-specific mortality data published in official statistics;
- examine how the prevalence of particular causes of death in the mortality structure changes across Russian regions; and
- identify the most obvious discrepancies across different regions.

Using the available tools and data, we provide an indirect estimation of the uniformity of cause-of-death coding practices across Russia, and seek to identify the most problematic points of disagreement between different regions. Furthermore, we present a broader picture of the quality of cause-of-death coding practices at the subnational level in Russia.

## Data and methods

### Regional data on causes of death

Regional death counts and mid-year population estimates by sex and age were obtained from the Russian Federal State Statistics Service. Age-standardized death rates (SDRs) for both sexes combined were calculated with the European Population Standard [43].

We use data for the period from 2002 (the year when RC-1999 was de facto implemented throughout Russia) to 2012 for a sub-sample of 52 Russian regions. To avoid the random fluctuations caused by small numbers of death events, we have limited our analysis to the 52 regions of Russia in which the annual population exposure (average for the period) was one million person-years or higher. We also excluded the Chechen Republic because death counts for this territory were only available from 2004 onward. This sample of 52 regions is presented in Table 2. In 2002–2012, 88.4 % of the total population and 88.5 % of all deaths in Russia were in these regions.

**Table 2:** Regions under study, by federal district of Russia

Central Federal District	Belgorod Oblast, Belgorod Oblast, Vladimir Oblast, Voronezh Oblast, Ivanovo Oblast, Tver Oblast, Kaluga Oblast, Kursk Oblast, Lipetzk Oblast, the city of Moscow, Moscow Oblast, Ryazan Oblast, Saratov Oblast, Smolensk Oblast, Tambov Oblast, Tula Oblast, Yaroslavl Oblast
Northwestern Federal District	Arkhangelsk Oblast, Vologda Oblast, the city of Sankt-Petersburg, Leningrad Oblast
Volga Federal District	Nizhny Novgorod Oblast, Kirov Oblast, Samara Oblast, Orenburg Oblast, Penza Oblast, Perm Kray, Ulyanovsk Oblast, Republic of Bashkortostan, Republic of Tatarstan, Udmurt Republic, Chuvash Republic
Southern Federal District	Krasnodar Kray, Astrakhan Oblast, Volgograd Oblast, Rostov Oblast
North Caucasian Federal District	Republic of Dagestan, Stavropol Kray
Ural Federal District	Sverdlovsk Oblast, Tyumen Oblast, Chelyabinsk Oblast, Khanty-Mansi Autonomous Area
Siberian Federal District	Altai Kray, Krasnoyarsk Kray, Irkutsk Oblast Kemerovo Oblast, Novosibirsk Oblast, Omsk Oblast, Tomsk Oblast, Zabaikalsk Kray
Far Eastern Federal District	Primorsky Kray, Khabarovsk Kray

For the same reason – i.e., to eliminate biases generated by small numbers – we assigned some items of the RC-1999 to broader diagnostic groups of causes of death. Moreover, we had to exclude some ICD-10 chapters from our analysis (Chapters III, VII, VIII, XII, XIII, XV) because the numbers of deaths from the causes that constitute these chapters were too low, and no meaningful grouping with the other chapters could be done. The final list of selected causes of death includes 70 items (Table 3).

**Table 3:** Causes of death under study

*N*	*Cause*	*RC*-*1999 codes*	*ICD*-*10 codes*
1	Tuberculosis and its sequelae	9-15, 54	A15-A19, B90
2	AIDS	44	B20-B24
3	Other infectious and parasitic diseases	1-8, 16–43, 45–53, 55	A00-14, A20-99, B00-19, B25-89, B91-99
4	Mouth and oropharynx cancers	56	C00-C14
5	Esophagus cancer	57	C15
6	Stomach cancer	58	C16
7	Colon and rectum cancers	60-61	C18-C21
8	Liver cancer	62	C22
9	Pancreas cancer	63	C25
10	Cancers of other digestive organs	59,64	C17, C23, C24, C26
11	Trachea, bronchus and lung cancers	66	C33-C34
12	Cancers of other respiratory, intrathoracic organs	65,67	C30-C32, C37-C39
13	Melanoma and other skin cancers	69-70	C43-C44
14	Mesothelial and soft tissue cancers	71	C45-C49
15	Breast cancer	72	C50
16	Cervix uteri cancer	73	C53
17	Corpus uteri cancer	74	C54-C55
18	Ovary cancer	75	C56
19	Prostate cancer	77	C61
20	Kidney cancer	79	C64
21	Bladder cancer	80	C67
22	Cancer of brain and central nervous system	82	C70-C72
23	Other cancers	68,76,78,81,83	C40-41, C51-52, C57-58, C60-63, C65-66, C68-69, C73-80, C97
24	Lymphomas and multiple myeloma	84-86	C81-C90, C96
25	Leukemia	87	C91-C95
26	Other neoplasms	89	D00-D48
27	Endocrine, nutritional and metabolic diseases	93-96	E00-E99
28	Mental and behavioral disorders	97-103	F00-F99
29	Diseases of the nervous system	104-111	G00-G99
30	Rheumatic diseases	115-116	I00-I09
31	Hypertensive diseases	117-120	I11-I15
32	Myocardial infarction	121-123	I21-I23
33	Atherosclerotic heart disease	125	I25.1
34	Other forms of ischemic heart diseases	127-129	I20, I24.1-9, I25.2-9
35	Pulmonary heart and circulation diseases	131	I26-I28
36	Other heart diseases	132	I30-I51
37	Subarachnoid hemorrhage	133	I60
38	Nontraumatic intracranial hemorrhage	135	I61-I62
39	Cerebral infarction	137	I63
40	Stroke, not specified as hemorrhage or infarction	139	I64
41	Other cerebrovascular disorders	141	I67-I69
42	Atherosclerosis	143	I70
43	Other diseases of arteries, arterioles, and capillaries	144	I71-I79
44	Disorders of veins and lymphatic vessels	145-146	I80-I89
45	Pneumonia	150-154	J12-J18
46	Chronic obstructive pulmonary disease	156-158	J40-J44
47	Other respiratory diseases	148-150, 154–155, 159-163	J00-11, J19-39, J45-99
48	Peptic ulcer disease	165-167	K25-K27
49	Alcoholic liver disease	173	K70
50	Fibrosis and cirrhosis of the liver	174	K74
51	Other diseases of liver	175	K71-K73, K75-K76
52	Diseases of pancreas	178	K85-K86
53	Other digestive diseases	168-172, 176–177, 179	K00-24, K28-K69, K80-84, K87-93
54	Nephritis and nephrosis	185-189	N00-N15
55	Other urinary diseases	190-191	N16-N39
56	Conditions originating in the perinatal period	206-216	P05-P96
57	Congenital malformations	217-225	Q00-Q99
58	Senility	226	R54
59	Other ill-defined and unspecified causes	227-228	R00-R53, R55-R99
60	Road traffic accidents	239-241, 272-274	V01-V99
61	Alcohol poisoning	247	X45
62	Other accidental poisoning	248	X40-44, X46-X49
63	Falls	242	W00-W19
64	Fires	246	X00-X09
65	Drowning	243	W65-W74
66	Accidental inhalation	244	W75-W84
67	Other unintentional injuries	245, 254-255	W20-W64, W85-W99, X10-X39, X50-X59, Y35, Y85-X89
68	Suicide	249	X60-X84
69	Homicide	250	X85-Y09
70	Injuries with undetermined intent	251	Y10-Y34

## Methods

To estimate the inter-regional variability of mortality from specific cause of death we used the cause-specific share of the all-cause age-standardized death rate:

1 $$ {S}_{r,c,t}=\frac{SD{R}_{r,c,t}}{SD{R}_{r,t}}100\%, $$

where *SDR*_*r*,*c*,*t*_ is the age-standardized death rate for cause *c* in region *r* in year *t*, and *SDR*_*r*,*t*_ is the all-cause age-standardized death rate in region *r* in year *t*. We used the indicators *S*_*r*,*c*,*t*_ instead of the cause-specific rates in order to eliminate the influence of variation in overall mortality levels across regions and over time.

Next, for each possible combination region/cause we calculated the indicator measuring the deviation from the cross-regional mean (period average) (2):

2 $$ {V}_{r,c}=\frac{1}{T}{\displaystyle {\sum}_{t=1}^T\left|\frac{S_{r,c,t}-\overline{{S}_{\bullet, c,t}}}{S_{\bullet, c,t}}\right|}\;100\%, $$

where $\overline{{S}_{\bullet, c,t}}$ is the mean of regional ${S}_{r,c,{t}^1}$, *T* – the length of time series. We thereby obtained a data set of scores in which each percentage score *V*_*r*,*c*_ shows how much on average (with respect to time) the share of cause *c* in the all-cause SDR of region *r* differs from an average of the inter-regional share of the same cause. The total size of the data set is equal to the number of regions multiplied by the number of causes of death.

### Visualization

After computing indicators *V*_*r*,*c*_ according to equation (2), we obtained a matrix that had 52 columns (the number of regions) and 70 rows (the number of causes of death). To present this matrix in an intelligible form, we plotted a heatmap in which each row corresponds to a particular cause of death and each column represents a specific region. The cells are colored based on the values of *V*_*r*,*c*_ using the yellow-red gradient palette. The points with a light yellow color have the lowest levels of deviation, and the points become darker in color as the degree of deviation increases. We set up our system of color gradation so that only the cases that deviated significantly from the average are clearly detectable on the heatmap. Deviations of less than 40 % from the average are not obviously recognizable on the heatmap, and are seen as low values. We used this color gradation deliberately in order to identify the cases for which the degree of deviation is so high that it is likely that they are attributable to differences in coding practices, rather than to real differences in regional epidemiological patterns.

### Statistical analysis of variability

To determine whether there is a certain regularity in the causes and the regions that are more likely than others to deviate from an average inter-regional level, we applied a least squares regression model (3) with two sets of dummy variables for regions and for causes of death:

3 $$ {V}_{r,c}=a+{b}_r{I}_r+{d}_c{I}_c+{\upvarepsilon}_{r,c} $$

where *a* is a constant term; *I*_*r*_ and *I*_*c*_ are, respectively, independent regional and cause-specific dummy variables; *b*_*r*_ and *d*_c_ are the coefficients on these variables; and ε_*r*,*c*_ is an error term.

We used “Kaluga Oblast” as the reference category for variable *I*_*r*_, and “trachea, bronchus, and lung cancers” as the reference category for variable *I*_*c*_. This region and this group of causes were chosen as the omitted reference units as these categories appeared to deviate from the average less than the others on our heatmap. Thus, the total number of estimated coefficients through a regression equals 120 (51 for regional and 69 for cause-specific dummies). We then checked the sustainability of these results using sensitivity analysis.

## Results

Before presenting our results, we will briefly describe a finding that was obtained outside of the framework of the current study. The initial impulse to conduct this study arose from a finding that emerged while we were engaged in a reconstruction of coherent cause-specific mortality time series in Russia. The introduction of the ICD-10 and the RC-1999 classification systems in 1999 resulted in inconsistencies between the mortality series coded under the RC-1999 and the series coded under the previous SC-1988 classification. Before we could compare mortality over a time period that was covered by several classifications, we had to reconstruct the cause-specific data series so that the full period was covered by the same classification. The reconstruction process was done using the method developed by J. Vallin and F. Meslé (for a description of the original method, see: Vallin and Meslé, 1988; Meslé et al., 1992; Meslé and Vallin, 1996) [44–46]. While performing this work, we discovered indirect indications that there could be significant discrepancies in cause-of-death coding practices across subnational entities in Russia. First, we found that the transitions to the ICD-10 and the RC-1999 classification systems at the regional level were not done simultaneously: four regions (the city of Moscow, Stavropol Kray, the Republic of Ingushetia, and the Sverdlovsk Oblast) postponed the transition for up to three years. While all of the deaths in the aforementioned regions were formally published in the official statistics under the new RC-1999 starting with the year after the transition, these deaths had been originally coded under the previous SC-1988 classification, and were then roughly translated into the items of the new RC-1999 classification. Second, we found that even after all of the regions had introduced the new classification, there were still significant regional disparities for some causes of death, many of which persist up to the present day.

These observations made during our reconstruction work, together with concerns raised in previous research literature about the quality of cause-of-death statistics in Russia, provided us with the starting points for our study. We realized that there was a need to identify and systematize the problems in the reporting of the underlying causes of death at a subnational level in Russia.

**Fig. 1:**
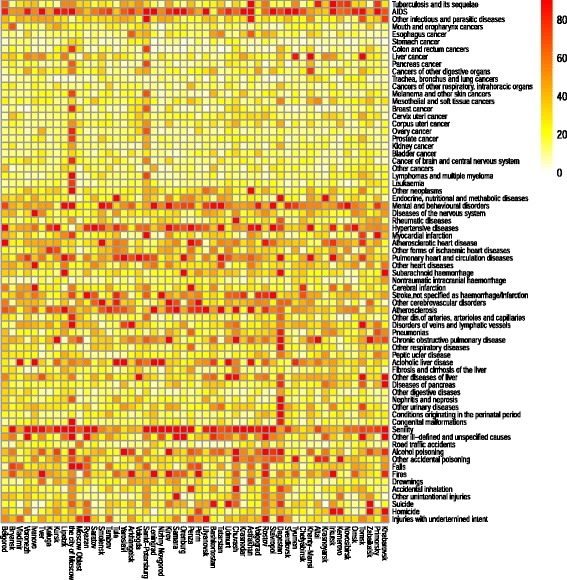
Heatmap on inter-regional variability in causes of death. Each row corresponds to a particular cause of death and each column represents a region. The cells are colored according to the values of V_*r*,*c*_

We turn now to the results directly obtained using the methods described in the “Methods” section. The heatmap presents the entire range of the *V*_*r*,*c*_ values in a transparent and observable form (Fig. 1). When looking at the heatmap, we can clearly see horizontal patterns that indicate that the causes of death vary greatly across the regions. We can also see some vertical patterns that show that certain regions have cause-specific mortality structures that deviate from the average more than those of other regions.

Table 4 provides the fragment of the regression results of the model (3). The coefficients *b*_*r*_ indicate to what extent an average of the scores *V*_*r*,*c*_ calculated for region *r* deviates from the omitted category “Kaluga.” Similarly, the coefficients *d*_*c*_ indicate to what extent the average of scores *V*_*r*,*c*_ for a certain cause of death *c* varies from the average for the omitted category “trachea, bronchus, and lung cancer.” We verified the robustness of the estimations with a sensitivity analysis that used different regional samples and changed the measure of inequality (relative root-mean square error instead of mean relative absolute error). The sensitivity analysis confirmed that our results have a high level of sustainability.

**Table 4:** Estimates of the regression coefficients of the OLS model (3)

*Region*	*Coef. b*_*r*_ (*95* % *CI*)	*p*-*value*	*Cause of death*	*Coef. d*_*c*_ (*95* % *CI*)	*p*-*value*
Sverdlovsk	−2.32 (−11.18, 6.54)	0.61	Stomach cancer	−0.67 (−10.94, 9.61)	0.90
Chelyabinsk	−2.31 (−11.17, 6.55)	0.61	Trachea, bronchus and lung cancers	[Ref]	—
Novosibirsk	−1.99 (−10.85, 6.87)	0.66	Kidney cancer	2.93 (−7.34, 13.21)	0.58
Moscow Oblast	−1.45 (−10.31, 7.41)	0.75	Breast cancer	2.98 (−7.29, 13.26)	0.57
Omsk	−0.87 (−9.73, 7.99)	0.85	Colon and rectum cancer	3.21 (−7.07, 13.49)	0.54
⁞	⁞	⁞	Cancer of other and ill-defined respiratory and intrathoracic organs	4.00 (−6.28, 14.28)	0.45
Kaluga	[Ref]	—	⁞	⁞	⁞
⁞	⁞	⁞	Atherosclerotic heart disease	34.02 (23.74, 44.3)	<0.001
Astrakhan	11.5 (2.64, 20.36)	0.01	Other symptoms and signs	34.16 (23.88, 44.43)	<0.001
Samara	11.7 (2.84, 20.56)	0.01	Other diseases of liver	34.23 (23.95, 44.51)	<0.001
Rostov	12.12 (3.26, 20.98)	0.01	Stroke not specified as hemorrhage or infarction	36 (25.72, 46.28)	<0.001
Tomsk	12.53 (3.67, 21.39)	0.01	Alcohol poisoning	36.32 (26.04, 46.6)	<0.001
Lipetzk	13.58 (4.72, 22.44)	<0.001	Fires	36.74 (26.46, 47.02)	<0.001
Chuvash	16.74 (7.88, 25.6)	<0.001	Alcoholic liver disease	40.8 (30.52, 51.08)	<0.001
Saint Petersburg	19.93 (11.07, 28.79)	<0.001	Chronic obstructive pulmonary diseases	41.83 (31.55, 52.1)	<0.001
City of Moscow	29.78 (20.93, 38.64)	<0.001	Pulmonary heart disease and diseases of pulmonary circulation	43.29 (33.01, 53.57)	<0.001
Dagestan	32.69 (23.83, 41.55)	<0.001	Hypertensive diseases	51.4 (41.12, 61.68)	<0.001
			Atherosclerosis	53.76 (43.48, 64.04)	<0.001
			Mental and behavioral disorders	63.14 (52.86, 73.41)	<0.001
			Senility	70.88 (60.6, 81.16)	<0.001
			AIDS	71.37 (61.09, 81.65)	<0.001

The regions and the causes of death with the lowest/highest variability are presented

Among the 69 causes of death that were assigned dummy variables, 45 causes showed a statistically significant (*p* < 0.05) deviation from the reference level, and 38 causes showed a deviation of *p* < 0.01. For 25 of these causes the deviation predicted by the model was higher than 20 %. The highest regression coefficients *d*_*c*_ were found for dummy variables corresponding to AIDS (+71.4 %), senility (+70.9 %), mental and behavioral disorders (+63.1 %), atherosclerosis (+53.8 %), hypertensive diseases (+51.4 %), pulmonary heart and circulation diseases (+41.8 %), chronic obstructive pulmonary diseases (+41.8 %), and alcoholic liver disease (+40.8 %).

The highest levels of consistency (the lowest regression coefficients *d*_*c*_) across regions were found for causes that represent different groups of cancers (from +0.7 % for stomach cancer to +11.8 % for the item “cancers of other digestive organs”). The other causes that deviated relatively little from the reference category were nontraumatic intracranial hemorrhage (+4.3 %) and transport accidents (+4.4 %).

**Fig. 2:**
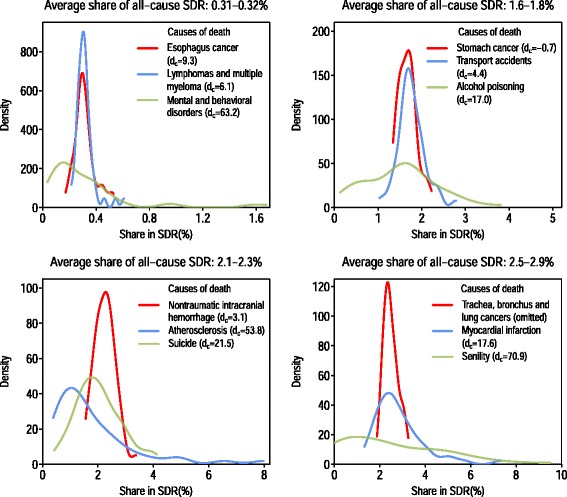
Examples of distributions of cause-specific shares of the all-cause SDR across 52 regions

Four panels of Fig. 2 present examples of distributions of causes of death that were very similar in terms of their contributions to overall mortality (period and regional average $\overline{{S}_{\bullet, c,\bullet }}$); however, the respective coefficients *d*_*c*_ returned by the regression model (3) for these causes differed substantially.

The distributions presented were dissimilar with respect to their kurtosis and skewness. In particular, the fourth panel illustrates three causes of death that were almost equal by average $\overline{{S}_{\bullet, c,\bullet }}$ (2.5 % for trachea, bronchus, and lung cancers; 2.6 % for senility; and 2.9 % for myocardial infarction). But the regional distributions of $\overline{{S}_{r,c,\bullet }}$ values were very different for these three causes. The range of variation (the difference between the maximum and the minimum regional values of $\overline{{S}_{r,c,\bullet }}$ for trachea, bronchus, and lung cancers was only 1.4 % (with the minimum in Smolensk equal to 1.9 %, and the maximum in Altay equal to 3.3 %), while the range of variation for myocardial infarction was 5.7 % (from 1.3 % in Lipetzk to 7.0 % in Primorsky) and the range of variation for senility was 8.8 % (from 0.0 % in four regions in the sample to 8.8 % in Voronezh). All of these distributions had very different levels of skewness as well. The regional values of $\overline{{S}_{r,c,\bullet }}$ for trachea, bronchus, and lung cancers were distributed almost symmetrically, with the modal value being approximately equal to the mean of the distribution. The distribution for myocardial infarction was highly skewed with a long right tail. Finally, the indicators $\overline{{S}_{r,c,\bullet }}$ for the item “senility” were almost uniformly distributed across all regions.

Different approaches in the reporting of senility as the underlying cause of death undoubtedly affected regional mortality rates from the other, more specific causes. The prevalence of the other “garbage codes”^1^ included in our analysis – i.e., the items “other ill-defined and unspecified causes” and “injuries with undetermined intent” – also varied considerably across regions (+34.2 and +28.5 compared with the reference category, respectively). Among the 52 regions under study, the average share of garbage codes combined was 7.0 % (period average for 2002–2012). In four of the regions the share of garbage codes was less than 3 %, while in 10 other regions the share was between 3 % and 5 %. However, in eight regions these causes contributed to overall mortality in more than 10 % of the cases, with the maximum contribution level of 15.2 % found in Ryazan.

Although the regional pattern on the heatmap is less apparent than the pattern for causes of death, some vertical structures are still clearly recognizable. The results of the least squared regression model demonstrate that in 13 regions the average of *V*_*r*,*c*_ values predicted by equation (2) were statistically different from the value in the reference (Kaluga), at the *p* < 0.05 level; and that in seven regions the values were statistically different at the *p* < 0.01 level. For nine regions, the average deviation was higher than 10 %, and was thus higher than in Kaluga; the top scores were found for Dagestan (+32.6 %), the city of Moscow (+29.8 %), and the city of Saint Petersburg (+19.9 %). In eight regions the coefficients *b*_*r*_ were negative, but none of those coefficients was statistically significant.

It is worth noting that there is no apparent spatial regularity in the distribution of the *V*_*r*,*c*_ scores across the regions. For instance, the city of Moscow, which had an estimated regression coefficient of *b*_*r*_ = 29.8; is surrounded by the Moscow Oblast, which had the same coefficient equal to 1.5.

**Fig. 3:**
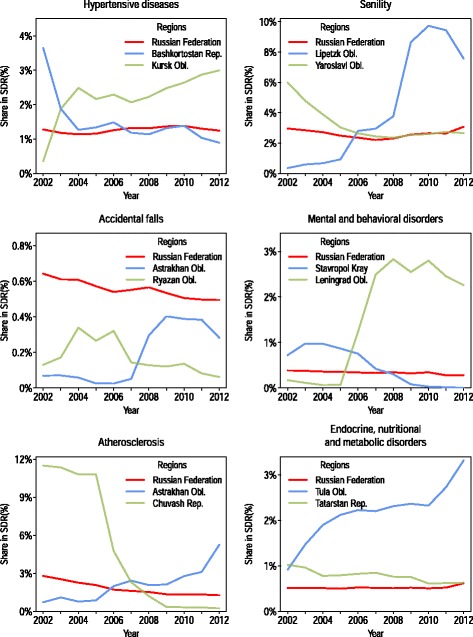
Examples of rapid and contrasting changes in regional cause-specific shares of all-cause SDR (both sexes combined). The trend for Russia as a whole is provided for comparison

The *V*_*r*,*c*_ scores we used for the analysis of spatial variability showed average (for the period) levels of deviation, but they did not indicate the sign (positive or negative) of the deviation or how the magnitude of the deviation changed over time. To find out whether the patterns of deviations were stable in the regions over an observation period, we inspected the regional time series. We found a number of regional cause-specific series that were unexpectedly distorted during the period 2002–2012. These abrupt and/or unpredictably large changes in mortality levels from particular causes over time may indicate a modification of coding practices, whereby some number of deaths that would have previously been coded to a certain item started to be coded to another item (Fig. 3).

Interestingly, the breaks in the regional time series occurred at different points in time, and the directions of these changes were sometimes even reversed in different regions. It therefore seems unlikely that regions introduced new coding practices in order to meet some baseline standards. Unpredictably large shifts in cause-specific series were not common; they took place in only a few regions and cannot be visually detected at the national level. However, when considering the small fluctuations in mortality trends by cause that can be observed at the national level, it is important to be aware that some of the changes may reflect changes in coding practices at the subnational level.

The most significant and numerous changes in regional trends for causes of death were found for AIDS, senility, mental and behavioral disorders, and atherosclerosis. The highest levels of stability over time were observed for different groups of cancers, nontraumatic intracranial hemorrhage, and transport accidents. Hence, there is an evident intersection between the causes of death with high levels of spatial variability and the causes of death for which the regional trends show a high degree of volatility. Similarly, the causes with the smallest degrees of variation across regions showed the highest levels of stability over time.

## Discussion

Like the Soviet system on which it is based, the current Russian system for producing information on causes of death is decentralized. The extent of this decentralization has increased substantially since the country made the transition to a new system of cause-of-death coding in 1999. Before the transition, the network of regional Statistics Offices had been responsible for coding the underlying cause of death; but since 1999, this task has been delegated to the individual medical practitioners. This shift coincided with the transition to the ICD-10. The Statistics Service coders had to code the underlying cause of death in accordance with the Soviet Abridged Classification, which offered them only 184 diagnostic items to choose from. By contrast, medical practitioners now have to assign the cause using the complete ICD-10 classification, which contains over 10,000 nosological items.

According to some experts, this change led to a deterioration of the Russian system of coding and gathering information on the causes of death, in part because no unified training in cause-of-death coding for medical workers was provided [47]. Moreover, medical professionals were not even given any centralized instructions for filling in medical death certificates and coding the causes of death in accordance with ICD rules [48]. This lack of preparation has led to specific difficulties with and discrepancies in coding practices across subnational entities and over time.

The present study has identified several problems with the cause-specific mortality statistics across the Russian territories.

We have found that while certain causes of death (e.g., cancers, transport accidents) have roughly comparable cause-specific shares across the regional mortality structures, there is a much greater degree of inconsistency in the prevalence of other cause-specific shares across the regions. For some causes, the magnitude of these inconsistencies is too large, and is therefore more likely to be artificial than to be indicative of natural variation across regions. Thus, it is possible that the regional differences in mortality from these causes reflect variation in coding practices, rather than real differences in the prevalence of diseases.

**Fig. 4:**
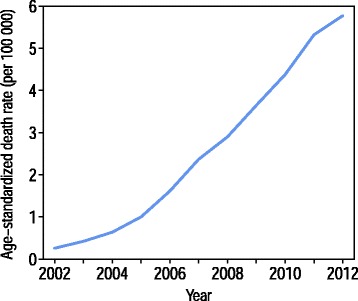
AIDS, standardized death rates per 100,000 (Russia, both sexes)

The lowest levels of consistency among the causes of death we investigated were found for AIDS. However, the high degree of variability of AIDS diagnoses cannot be regarded as a problem of coding accuracy only. AIDS was a new cause of death at the start of our study period, and the number of people who were dying from this disease was clearly increasing as the period progressed. Over time, our understanding of and ability to detect the disease have improved, and coding practices have adapted accordingly. Mortality from AIDS has been rising rapidly in Russia over the last decade (Fig. 4). One piece of evidence that supports the claim that there is “natural” wide variation in AIDS mortality across different regions of Russia is the finding that there is a strong positive correlation between the registered prevalence of HIV in a given region [49] and the share of AIDS in the regional all-cause SDR (the correlation was 0.88 in 2012).

As is the case for other communicable diseases, AIDS has spread unevenly across the population. Some regions could be a nidus of infection, while others have had much lower incidence levels. Thus, the large degrees of spatial and temporal variation in the contributions of AIDS to overall mortality can be explained. However, some portion of the variation in the prevalence of AIDS mortality across Russian regions may have also been caused by discrepancies in the cause-of-death coding practices. In many countries, deaths from AIDS are systemically miscoded under tuberculosis, endocrine disorders, Kaposi’s sarcoma, meningitis, encephalitis, certain garbage codes, and other causes of death [50, 51]. C. Murray and co-authors estimated that the real number of deaths from AIDS in Russia in 2013 was 16,138 (95 % uncertainty interval 11,963 to 22,526) [51], or 52 % higher than the number that was officially reported by the Russian State Statistics Service.

In 2010, E. Tzybikova examined 6249 deaths that occurred among patients with newly diagnosed tuberculosis in 80 Russian regions. She found that in 61 regions some deaths from AIDS were mistakenly coded as tuberculosis. While tuberculosis was chosen as an underlying cause of death, AIDS was listed as an associated cause, which violates the ICD instructions for sequencing the causes of death [52]. The total number of such cases found by this study was 1,004. When we look at the 6784 deaths from AIDS recorded in the official statistics in 2010, it appears that a very significant fraction of the deaths were misclassified. Unfortunately, this study did not provide a detailed explanation of the study design, and did not investigate the regional peculiarities in the misclassification of AIDS and tuberculosis. However, the finding that there were incidents of misclassification in 61 of the 80 regions studied may indicate that the extent of the misclassification of deaths from AIDS also differs across regions.

Other groups of infectious diseases that we studied, such as “tuberculosis” and “other infectious diseases,” had medium levels of inconsistency compared to other causes of death. As was shown above, regional variation in mortality from tuberculosis can be affected by the misclassification of AIDS. Difficulties in certifying deaths with AIDS/tuberculosis co-infection are common, especially in countries with a high burden of HIV [50, 53]. Nevertheless, it should be noted that during the period of observation the prevalence of tuberculosis in the mortality structure of Russian regions ($\overline{{S}_{\bullet, c,t}}$ is equal to 1.3 %) was several times higher than mortality from AIDS ($\overline{{S}_{\bullet, c,t}}$ is equal to 0.2 %). Hence, the miscoding of these two causes distorts the mortality statistics for AIDS much more significantly than for tuberculosis.

While high levels of spatial and temporal heterogeneity are normal in the transmission of infectious diseases, having to rely solely on the data reported by official statistics makes it difficult to determine whether high degrees of variation in mortality from infectious causes reflect real differences in the prevalence of disease, or are indicative of differences in coding practices as well. But for causes of death from non-communicable diseases, it seems rather unlikely that very high levels of within-country variation are natural. Our finding that some non-communicable diseases had much higher levels of spatial variation than some communicable diseases can serve as indirect proof that the level of variation we found for some non-communicable diseases is too high and cannot be accurate.

Very low levels of consistency were found for some groups of causes from the ICD chapter “diseases of the circulatory system.” For some of these causes, the level of consistency would have been greater if we had assigned them to broader groups of items. For instance, within the group of ischemic heart diseases the ratio between the inter-regional maximum and the inter-regional minimum values of *V*_*r*,*c*_ calculated according to equation (2) amounts to 13.1 for “atherosclerotic heart disease,” 5.6 for “myocardial infarction,” and 4.3 for “other forms of ischemic heart diseases.” But if we combine all of these items into one group, “ischemic heart disease,” this ratio would be only 3.1. Similar results can also be obtained for the group of “cerebrovascular diseases.” These lower levels of inconsistency at higher levels of aggregation suggest that conflation often occurs when the possible causes of death are medically similar. Analyzing cause-specific mortality at higher levels of aggregation can reduce biases.

Coding discrepancies can undermine cause-specific analysis more significantly for causes that cannot be meaningfully grouped together with other items. Categories that represent complete ICD chapters, such as “diseases of the nervous system,” “endocrine, nutritional, and metabolic disorders,” and “mental and behavioral disorders” had very high levels of spatial and temporal inconsistencies in our analysis. Even more biases in the analysis of cause-specific mortality are caused by spatial and temporal differences in the use of garbage codes from the ICD-10 chapter XVIII, “symptoms, signs, and abnormal clinical and laboratory findings;” or groups of causes, such as “injuries of undetermined intent.” The propensity to assign garbage codes as underlying causes of death varied significantly across Russian regions. As garbage codes constitute a high share of the causes of death recorded in the Russian mortality structure, regional and period discordances can heavily affect the comparability of mortality indicators for other specific groups of causes of death that are misclassified with garbage codes.

In terms of spatial variations, a few regions can be pinpointed as having the cause-specific mortality structures that deviate the most starkly from the inter-regional average: the cities of Moscow and Saint Petersburg, which are constituent federal units; and the Republic of Dagestan, a Muslim region located in the North Caucasus. We offer several hypotheses for why these particular regions had the highest scores in our analysis.

First, these three regions have the lowest overall mortality levels of the 52 regions in our sample. Lower mortality levels are generally indicative of certain mortality structures. In particular, lower mortality levels usually correspond with a higher share of deaths from neoplasms in relation to other causes of death. Accordingly, it is quite apparent on the heatmap that there are substantial differences between Moscow and Saint Petersburg on the one hand and the other Russian regions on the other in terms of the share of deaths from neoplasms relative to overall mortality. But the deviating pattern for Dagestan is mainly attributable to the relative shares of other causes of death. It is important to note that the Republic of Dagestan differs considerably from the other regions in our sample, as it is the only national republic of North Caucasus selected for this analysis, and the Muslim regions of North Caucasus have much lower mortality levels from alcohol-related causes than the rest of Russia. In addition, in these regions there are long-term concerns about the understatement of mortality at infant and old ages due to the underreporting of deaths, and about the overstatement of age [54, 55].

Second, the populations of the cities of Moscow and Saint Petersburg are entirely urban. Dagestan, by contrast, is the only region in the sample in which the urban population is still smaller than the rural population. Therefore, it is possible that the significant differences in the mortality structures between these three regions and the other regions of Russia are at least partly attributable to the differences between urban and rural populations.

The other possible explanation is a registration effect. A death in Russia can be registered either at the location of the deceased’s permanent residence or at the location of death. This may result in certain biases in mortality statistics at the regional level, which can be especially large for Moscow and Saint Petersburg. First, there are a number of large federal medical centers in these two cities that specialize in the treatment of specific diseases and especially of cancers. In addition to residents of Moscow and Saint Petersburg, residents of other regions may be treated in these centers. Among all deaths from cancers in Moscow in 1990–1994, 4.8 % of the men and 5.6 % of the women who died were non-residents [56]. Additionally, Moscow and Saint Petersburg have huge migration inflows. The cause-specific mortality structures of these cities may therefore be affected by the selectivity in the health status of arriving migrants. Arkhangelsky and co-authors found that the cause-specific mortality structures of residents and non-residents in Moscow are very different [57]. Non-residents are, for example, more likely than residents to die from external causes, infectious diseases, and ill-defined conditions.

The results obtained in our study suggest that a complex series of actions will be needed to standardize regional approaches to cause-of-death coding and to improve the comparability of cause-specific mortality data within Russia. These actions should focus on strengthening the legal and regulatory framework for mortality statistics, improving the quality of human resources, and ensuring the full implementation of ICD standards. A national “gold standard” of training on death certification should be developed for medical practitioners. To increase the likelihood that medical workers will adhere to a uniform set of coding principles, the training procedures should be standardized to the greatest possible extent. Ideally, an automated, centralized coding and/or training software application would be designed and implemented across the country. The regular monitoring of the comparability of cause-specific mortality data reported by regions is also essential. In our study we took the average region/cause deviations for an 11-year period; thus, only the long-term deviations from the inter-regional average level were highlighted. Surprisingly, the number of such long-term deviations was found to be quite large. This finding suggests that regions can follow different coding practices for a long period without these discrepancies being discovered by the responsible federal authorities. Additional checks must be carried out in cases in which mortality from a certain cause in a certain region obviously deviates disproportionately from the average level, and the origins of these kinds of deviations should be thoroughly investigated.

We also suggest producing an aggregated list of causes of death that can be used in analyses of regional mortality patterns with a minimal risk of inter-regional incomparability and biases. Such a list should be regarded exclusively as a stopgap measure. Developing and implementing a national plan for strengthening the quality of cause-of-death statistics is essential, and should still be seen as the highest priority. But as making substantial improvements takes time, in the interim the aggregated list can be useful for analyzing cause-specific mortality in Russia at the regional level.

### Limitations

Our study has several limitations. The first arises from the indirect character of the method proposed. As we analyzed the official cause-specific mortality data as they are, we can make only indirect assessments of the quality and validity of these data. Although we can observe spatial and temporal variations in cause-specific shares, we cannot be certain whether they are caused by real mortality differences or by discrepancies in coding practices. While we can be reasonably sure that such discrepancies are present when the regional deviations in the causes of death are especially large, we cannot judge the less obvious cases. Further research is needed to determine why there are problems in the data.

The second limitation follows from the grouping of causes of death in the Russian Abridged Classification. It is impossible to extract from these data certain groups of ill-defined cancers and ill-defined cardiovascular diseases, which are also regarded as so-called garbage codes [58–60]. In most cases, such garbage codes are combined in the RC-1999 with some well-defined codes under the heading “other and unspecified.” For instance, the group “cancers of other and independent (primary) multiple sites” in the RC-1999 includes, in addition to the codes for ill-defined cancers (C76, C80, C97), codes that correspond to neoplasms with specific localization, and that cannot be referred to as garbage codes, such as “cancer of eye and adnexa (C69)” and “cancers of thyroid and other endocrine glands (C73-C75).” Because these codes are ill-defined, we could not compare their prevalence across the regions, yet this is an important criterion for evaluation the quality of cause-of-death coding [2].

Third, our study was based on death counts obtained at the regional level. However, while the coding procedure in Russia is performed in a completely decentralized manner at the level of medical practitioners, there may also be some important discrepancies within the regions themselves.

## Conclusion

The systematic analysis we performed showed that there is a high degree of variance in the coding practices for some causes of death across Russian regions. We found that the mortality statistics for some causes are more reflective of the coding practices than of the real epidemiological situation. These problems of comparability can affect the validity and the generalizability of cause-specific mortality statistics. These possible biases should be taken into account when performing mortality analyses. There is an urgent need to improve the uniformity and the stability of coding practices at the subnational level in Russia, as doing so would strengthen the accuracy and the quality of mortality statistics.

## Footnotes

1 The term “garbage coding” was introduced by C. Murray and A. Lopez in 1996, and refers to the practice of assigning deaths to causes that do not meet the basic standard of generating useful information for public health analysis [58]. The prevalence of “garbage codes” in all-cause mortality structure is commonly used as one of the indicators for estimating the validity and reliability of mortality statistics [2, 59]. The overuse of garbage codes distorts mortality statistics from the other (“non-garbage”) causes of death [60].
